# Comparison of multiple imputation algorithms and verification using whole-genome sequencing in the CMUH genetic biobank

**DOI:** 10.37796/2211-8039.1302

**Published:** 2021-12-01

**Authors:** Ting-Yuan Liu, Chih-Fan Lin, Hsing-Tsung Wu, Ya-Lun Wu, Yu-Chia Chen, Chi-Chou Liao, Yu-Pao Chou, Dysan Chao, Ya-Sian Chang, Hsing-Fang Lu, Jan-Gowth Chang, Kai-Cheng Hsu, Fuu-Jen Tsai

**Affiliations:** a Center for Precision Medicine, China Medical University Hospital, Taichung, 40447, Taiwan; b Artificial Intelligence Center for Medical Diagnosis, China Medical University Hospital, Taichung, 40447, Taiwan; c Department of Medicine, China Medical University, Taichung, Taiwan; d Department of Neurology, China Medical University Hospital, Taichung, Taiwan; e Epigenome Research Center, China Medical University Hospital, Taichung, 40447, Taiwan; f Million-person Precision Medicine Initiative, China Medical University Hospital, Taichung, 40447, Taiwan; g Department of Medical Research, China Medical University Hospital, Taichung, 40402, Taiwan; h School of Chinese Medicine, China Medical University, Taichung, 40402, Taiwan; i Division of Pediatric Genetics, Children’s Hospital of China Medical University, Taichung, 40447, Taiwan; j Department of Biotechnology and Bioinformatics, Asia University, Taichung, 41354, Taiwan

**Keywords:** Imputation, SNP array, Whole genome sequencing, CMUH genetic biobank

## Abstract

A genome-wide association study (GWAS) can be conducted to systematically analyze the contributions of genetic factors to a wide variety of complex diseases. Nevertheless, existing GWASs have provided highly ethnic specific data. Accordingly, to provide data specific to Taiwan, we established a large-scale genetic database in a single medical institution at the China Medical University Hospital. With current technological limitations, microarray analysis can detect only a limited number of single-nucleotide polymorphisms (SNPs) with a minor allele frequency of >1%. Nevertheless, imputation represents a useful alternative means of expanding data. In this study, we compared four imputation algorithms in terms of various metrics. We observed that among the compared algorithms, Beagle5.2 achieved the fastest calculation speed, smallest storage space, highest specificity, and highest number of high-quality variants. We obtained 15,277,414 high-quality variants in 175,871 people by using Beagle5.2. In our internal verification process, Beagle5.2 exhibited an accuracy rate of up to 98.75%. We also conducted external verification. Our imputed variants had a 79.91% mapping rate and 90.41% accuracy. These results will be combined with clinical data in future research. We have made the results available for researchers to use in formulating imputation algorithms, in addition to establishing a complete SNP database for GWAS and PRS researchers. We believe that these data can help improve overall medical capabilities, particularly precision medicine, in Taiwan.

## 1. Introduction

A genome-wide association study (GWAS) can systematically analyze the contributions of genetic factors to a wide variety of complex diseases and to quantitative human traits and conditions such as height [1], body mass index [2], diabetes [3], cancer [4,5], and high cholesterol [6]. These types of studies have indicated new treatment pathways for those conditions, such as the 10 novel genetic single-nucleotide polymorphisms (SNPs) and 9 reported SNPs that were identified for risk of familial short stature [1]. GWAS reports have also provided evidence for previously suspected molecular mechanisms. In short, GWASs have considerably changed the analysis of human genetics in recent decades by providing a systematic method for gaining deeper insights into genetic diseases.

One limitation of such studies SNP genotyping arrays is that only a small component of human genetic variation is assayed, such as SNP [7]. Thus, detecting signals of association from rare variants is difficult. Whole-genome sequencing (WGS) with sufficient coverage can detect the rarest of mutations with remarkably high accuracy [8]. However, for screening large numbers of people, WGS services are prohibitively expensive. A more cost-efficient method of genotyping rare variants is to impute SNP array data [9].

Genotype imputation is commonly applied in GWASs [10]. Imputation methods entail combining a reference panel of SNP-genotyped individuals with a study sample collected from a genetically similar population and genotyped as a subset of these SNP sites [7]. Imputation algorithms predict unobserved genotypes in a study sample by using a population genetic model to extrapolate allelic correlations measured in the reference panel. Sophisticated imputation algorithms have been proven to provide clearer genetic information, which is helpful for design replication and fine-positioning research in GWAS study [11]. Imputation results can facilitate meta-analysis tasks because they enable combining data sets collected using genotyping chips from different sources for increased power [1,7,9,11]. Imputation results can also be used in the classification of HLA alleles [12] and pharmacogenetics- related gene research [13].

The problem of missing data is prevalent in statistics and in human genetic studies. However, conventional methods cannot solve a new type of imputation problem in GWASs; this problem involves an extremely high rate of missing data in genotyping results compared with the WGS data. Less than 1% of most GWAS genotyping data contain known genetic variants, and the remaining >99% of the data contain missing information on genetic variants that must be imputed [14]. Furthermore, common statistical imputation techniques, such as regression, do not model the key characteristics of genetic data. These challenges necessitate the development of statistical methods and computational tools created explicitly for genotype imputation in GWASs.

Several state-of-the-art algorithms are available for genotype imputation, including IMPUTE2 [15], IMPUTE4 [16], IMPUTE5 [17], and Beagle5.2 [18]. These imputation algorithms are mostly based on the hidden Markov model (HMM) implementation of the Li and Stephens model [19]. Although IMPUTE2 is more dated than the other aforementioned imputation programs, it can still achieve 99% accuracy with the 1000 Genomes Project reference panel [20]. IMPUTE4 can also improve the run time of an algorithm and was used in a large-scale imputation process for the UK Biobank study [16]. Both IMPUTE5 and Beagle5.2 contain their own compact reference panel formats designed to improve large-scale imputation run time and memory usage. In addition, IMPUTE5 utilizes the positional Burrows–Wheeler transform along with the HMM to increase the speed and scalability of the imputation of large reference panels [17].

These imputation algorithms have unique characteristics. Nevertheless, no study has compared their performance for the same data. Accordingly, in this study, we used data collected at China Medical University Hospital (CMUH) to conduct a comparison of these algorithms.

## 2. Methods

### 2.1. Data source

WGS data (for 1463 individuals) were obtained from the Taiwan Biobank (TWB) with the approval of the respective ethical committees of CMUH and the TWB (CMUH108-REC1-0910). The WGS data were sequenced using Illumina Hi-Seq 2500. Reads were mapped to the reference genome (hg38) by using the Burrows-Wheeler Aligner (BWA) [21], and variant calling was executed using GATK [22]. Finally, VEP was used for annotation [23,24]. All analysis parameters were set at their default values.

Additionally, we obtained the TWB customized SNP array data for all 1463 participants from the TWB to validate the accuracy of each imputation algorithm. We also collected 95 people WGS data items from the CMUH database. These WGS data were sequenced using Illumina NovaSeq 6000 and analyzed using the Illumina DRAGEN Bio-IT Platform (v3.6). We selected the DRAGEN DNA Pipeline to obtain the germline mutation variants. All parameters were based on the default value in DRAGEN.

### 2.2. Informed consent

The China Medical University Hospital Precision Medicine Project was initiated in 2018 and remains operational. This project was approved by the respective ethical committees of CMUH (CMUH107-REC3-058 and CMUH110-REC3-005). More than 170,000 people have contributed thus far.

### 2.3. Imputation workflow and experimental design

Before running the imputation programs, we first constructed a haplotype reference panel and pre-processed the SNP array data. Although WGS data from the 1000 Genome Project are widely used to assemble reference panels, Mitt et al. [25] and Wei et al. [26] have achieved highly accurate imputation results by using a population-specific reference panel. Accordingly, we used WGS data from the TWB (TWBWGS) as the reference to impute an SNP array that was specifically designed for the Taiwanese population. Developing the TWBWGS reference panel involved three main steps: ensuring quality control of reference variants, phasing those variants after quality control, and converting the TWBWGS haplotypes to the corresponding reference panel format for each imputation program. The preprocessing of SNP array data involved variant quality control and prephasing. The quality control step removed potential genotyping error variants, and the prephasing step could significantly accelerate the imputation run time. After preprocessing both the reference panel and SNP array data, we could run the imputation programs.

### 2.4. Preprocessing of imputation reference panel

The quality control procedures for WGS data were based on the conducted studies by Mitt et al. [25] and Wei et al. [26]. In addition to the WGS information, data for the East Asian (EAS) participants of the 1000 Genomes Project (HG38 phase 3) [27] were used to boost the imputation accuracy of IMPUTE2. For both WGS and EAS data, we used bcftools [28] to exclude variants with a minor allele count (MAC) of <3, variants with missing genotypes, variants other than SNP/INDEL, and multiallelic variants. In the EAS panel, we used vcftools [29] to exclude variants with a Hardy-Weinberg equilibrium of less than 1e-7 (−hwe 1e-7). Finally, the WGS data were phased using SHAPEIT2 [30]. After the variant quality control and phasing steps, the WGS data contained 15,471,490 variants and the EAS data contained 9,984,021 variants.

To validate the imputation accuracy, we randomly formed a group subset (100 individuals) from the WGS data for internal testing. The remaining WGS data (1363 individuals) were used to construct a reference panel for each imputation program. IMPUTE2 and IMPUTE4 require the hap/legend reference panel format, and the newer IMPUTE5 and Beagle5.2 support the vcf reference panel format. However, the developers of both IMPUTE5 and Beagle5.2 recommend using their own unique reference panel formats (imp5 and bref3, respectively) to optimize memory usage and run time. Consequently, we converted the WGS data into imp5 and bref3 formats.

### 2.5. SNP array data quality control

The SNP array was determined to contain approximately 714,457 SNPs. We used PLINK1.9 for this analysis [31]. We excluded samples and SNPs with missing rates (−geno 0.1 for SNPs and –mind 0.1 for samples). We filtered out variants with a Hardy–Weinberg equilibrium p value of <1e-6 (–hwe 1e-6) and minor allele frequency (MAF) of <1e-4 (–maf 0.0001). Therefore, 515,310 variants and 175,871 people passed the filters and the quality control process. Because our imputation reference panel was phased using SHAPEIT2, we used the same tool to prephase the SNP array data. In addition, we prephased the SNP array data with SHAPEIT4 to determine whether the newer haplotype estimation tool would produce the same imputation accuracy. The default parameters of both SHAPEIT2 and SHAPEIT4 [32] were applied, and reference WGS was used as the phasing reference.

### 2.6. Genotype imputation

All imputation programs, namely IMPUTE2, IMPUTE4, IMPUTE5, and Beagle5.2, were implemented using their default parameters, except for the effective population size and the buffer region. The effective population size (-ne) indicates the genetic diversity of the model; a large effective population size represents an extensive population of diverse individuals. For this study, we set the effective population size to 20,000 for all imputation programs. To reduce memory usage, all imputation programs impute small chunks of each chromosome separately and merge all the imputed chunks at the end of the process. The buffer region represents the number of bases that overlap between chunks; it was set to 500,000 bases in this study. All imputation programs were executed on the Azure Cloud HB120rs_v2 virtual machine with 120 vCPUs and 480 GiB of RAM. By using different combinations of multithreads and multiprocesses, we could impute each chunk in parallel to optimize the machine’s run time.

Because IMPUTE2 includes the feature of merging two different reference panels, we imputed the SNP array data based on two reference panels separately derived from the TWB and 1000 Genomes EAS WGS data. Other imputation programs only allow one reference panel; therefore, we used WGS data from the TWB reference panel for each program.

The accuracy of the imputation result was measured using BCFtools gtcheck [28] to assess the concordance rate between the imputed genotypes and the WGS data. The BCFtools gtcheck default error probability assumes 1 sequencing error in 10,000 genotypes. The parameter –error-probability was set to 0 to compare the discordance between imputed and validation genotypes.

## 3. Results

### 3.1. Established imputation models and comparisons between IMPUTE and Beagle

**Fig. 1:**
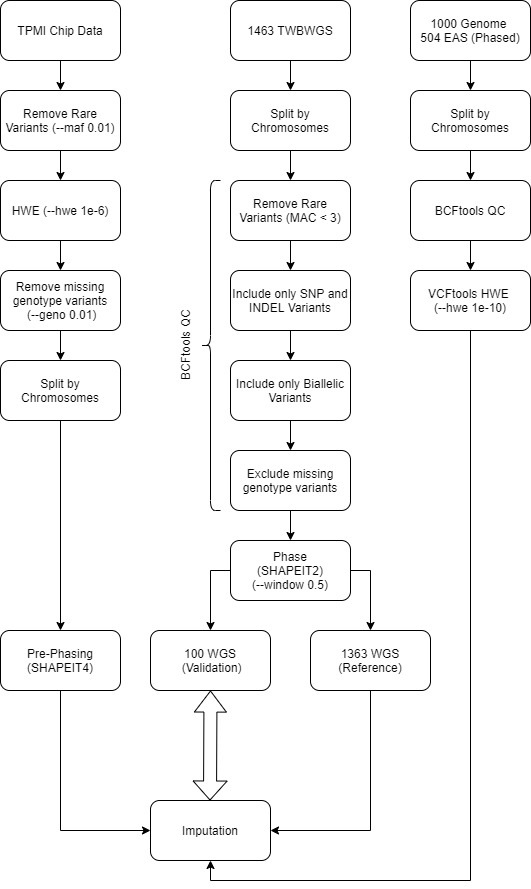
Overview of study pipeline. WGS data of TWB and EAS were used for model construction. For the TWB data, 100 WGS data items were in the validation cohorts and 1363 WGS data items were in the reference cohorts. For the 1000 Genome Project data, 504 EAS WGS data items were obtained.

We collected WGS and SNP array data from the TWB and downloaded the WGS data of the EAS participants in the 1000 Genome Project (HG38 phase 3). We used four algorithms (IMPUTE2, IMPUTE4, IMPUTE5, and Beagle5.2) and two reference bases (TWB and EAS) to construct our imputation model (Fig. 1). Our results revealed that Beagle5.2 exhibited the fastest calculation speed, smallest storage space, highest specificity, and highest number of high-quality variants. This algorithm required only 0.68 min per case to complete the imputation and only 1 GB of storage. Beagle5.2 made no extraneous imputations of SNPs, meaning that it displayed 100% specificity. Although the sensitivity of Beagle5.2 was slightly lower than that of the other algorithms, its accuracy was still up to 98.75%, and it could obtain the greatest number of high-quality variants (15,277,414) (Table 1).

**Fig. 2:**
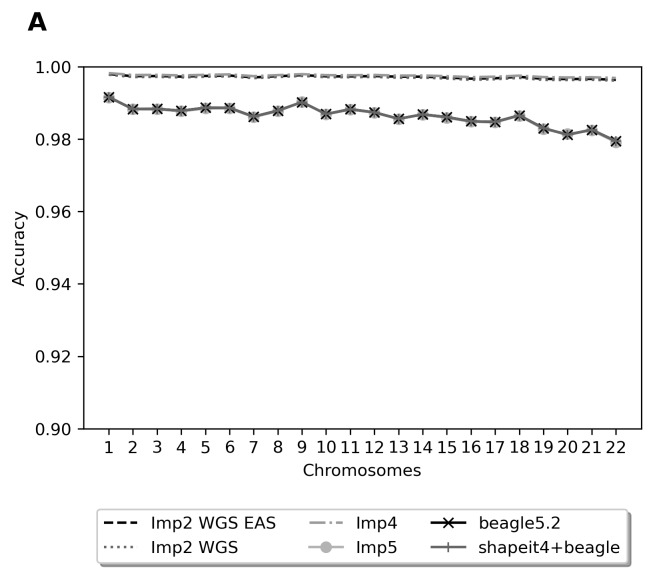
Imputation accuracy rates and number of imputed variants. (A) Accuracy breakdown of whole-genome imputation per chromosome for each imputation algorithm; (B) intersection of imputed genotype with WGS ground truth.

We also compared the algorithms in terms of their accuracy in each chromosome (Fig. 2A). Beagle5.2 exhibited the lowest accuracy on chromosome 22; nevertheless, its overall accuracy still reached 98.75%. The average accuracy on each chromosome was approximately 99.75% for IMPUTE2, IMPUTE4, and IMPUTE5. Furthermore, we examined the specificity of the different algorithms. Because extra variants were produced by IMPUTE2, IMPUTE4, and IMPUTE5 but not by Beagle5.2 (Fig. 2B), the specificity levels of IMPUTE2 (81.1%), IMPUTE4 (89.51%), and IMPUTE5 (99.5%) were lower than that of Beagle5.2 (100%; Table 1). Accordingly, we selected the Beagle5.2 algorithm to impute the CMUH SNP array data.

### 3.2. Imputation of SNP array data from CMUH by using Beagle5.2

**Fig. 3:**
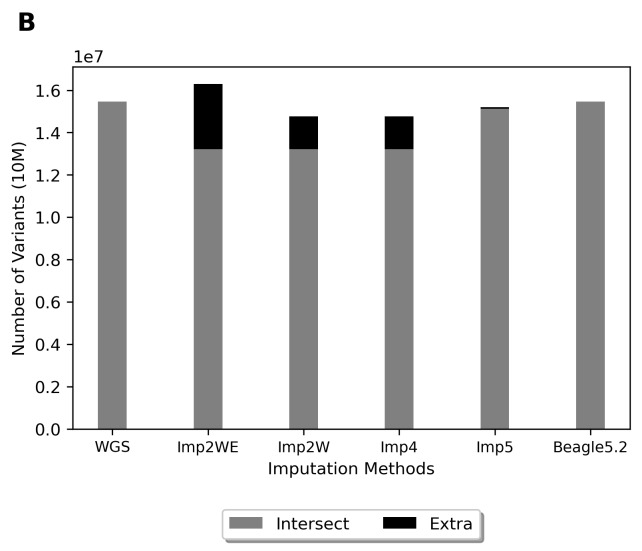
Variant distributions of MAF. (A) MAF of TPMv1 variants; (B) MAF of WGS reference panel variants; (C) MAF of imputed variants.

**Fig. 4:**
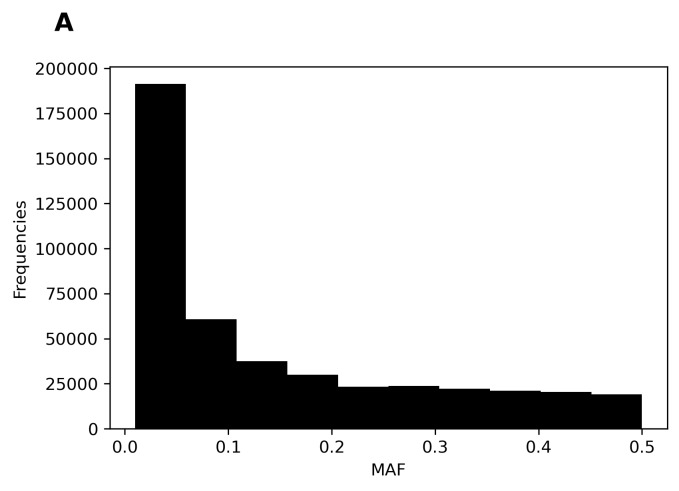
R^2^ and concordance of MAF. (A) R^2^ of imputed SNP array data; (B) concordance of imputed SNP array data. Horizontal axis represents MAF. The vertical axis represents R^2^ and concordance.

We collected genotyping data for 175,871 individuals from the CMUH genetic database. Before imputation, 515,310 variants passed quality control. The MAF for most variants was 0%–1% (Fig. 3A). The MAF for most variants in the TWB reference data was 0%–1% (Fig. 3B). After imputation, the distribution of the MAF in the imputation data was similar to that in the TWB reference data (Fig. 3C). We observed an *R*^2^ value of approximately 0.96 and concordance of 0.99–0.95. The *R*^2^ value exhibited an upward trend as the MAF increased (Fig. 4A). By contrast, the concordance exhibited a downward trend as the MAF increased (Fig. 4B). Finally, 15,277,414 variants passed quality control.

### 3.3. Use of external WGS to verify the results of the imputation

**Fig. 5:**
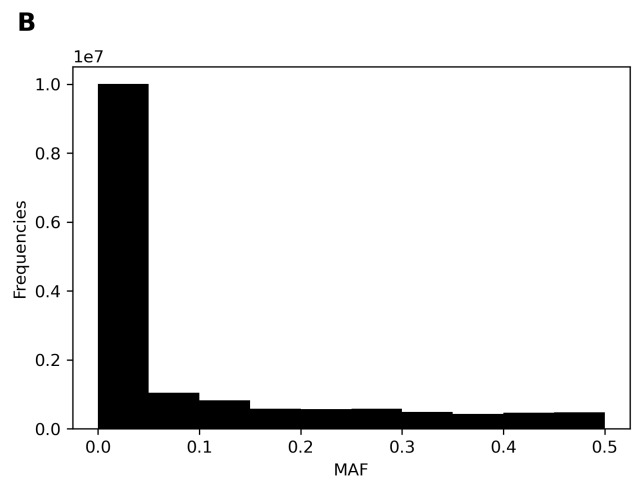
External WGS data for verifying the imputation results. Horizontal axis represents MAF. The left vertical axis represents mapping rate and accuracy for the line chart. The right vertical axis represents allele count for the graph.

We collected 95 WGS data from the CMUH genetic database. These WGS data were the germline mutation variants produced by Illumina DRAGEN. The imputed data were filtered out using an alternate allele dose of <0.3 and a genotype posterior probability of <0.9 as the criteria. We analyzed the mapping rate and accuracy in 95 samples. Overall, we observed a 79.91% mapping rate and 90.41% accuracy in our imputed variants. Most of the imputed variants had an MAF of >10%. Therefore, accuracy showed a downward trend as the MAF increased (Fig. 5).

In summary, we compared four imputation algorithms in this study. For timeliness and accuracy, we used Beagle5.2 to impute our SNP data. We obtained 15,277,414 high-quality variants from 175,871 samples. We also used external WGS to verify the imputation results. The verification results revealed a 79.91% mapping rate and 90.41% accuracy in our imputed variants. In future research, these results will ideally be combined with clinical data to assist in improving the provision of precision medicine in Taiwan.

## 4. Discussion

In recent years, the public health benefits of genetic research have been greatest at the population level. Most countries have been establishing their own genetic databases, such as the UK Biobank [16] and the Japan Biobank [33]. Ethnic specificity is critical in genetic research [34]. Accordingly, establishing a genetic database specific to Taiwanese society is essential. Before our study, no large genetic databases belonging to a single institution was available, although a genetic database integrating data from multiple institutions was already established [35]. We can efficiently combine our genetic database with more than 10 years of electronic medical records including clinical laboratory, image, diagnosis, operation, and hospitalization information [36]. Therefore, we can inexpensively and effectively execute genetic tests on participants while simultaneously collecting genetic profiles. The database can also be used for polygenic risk score (PRS) calculations for common diseases and for future GWASs [37].

The extremely high rate of missing data in genotyping results compared with the whole-genome data remains problematic. Specifically, <1% of most GWAS genotyping data contain known genetic variants, and the remaining >99% of the data contain missing information on genetic variation that must be imputed. We compared several common algorithms with unique advantages and limitations. The algorithm we selected as ideal was Beagle5.2. There was poorer accuracy Beagle5.2 algorithm than the other algorithm although there was the best specificity. If the unpaired SNPs were included in the error rate, Beagle5.2 will have the best accuracy. It possessed the most efficient computing speed and the highest specificity (Table 1) and was perfectly suitable for use with large-scale genetic databases.

In previous studies, few researchers have used WGS to verify the results of imputation [38]. In the present study, we used 95 WGS data to verify the results of the imputation. Even if the internal verification was as high as 98.75%, the accuracy was only 90.41% in the external verification. We also observed that the numbers of imputed variants were positively correlated with the MAF and that the matching rate was negatively correlated with the MAF. We observed almost no change in accuracy. We found that the accuracy (90.41%) of externally verified data was consistent with the GP (0.9) value (Fig. 5) [39]. The reason for the difference between internal and external verification is that the data provided by two different organization. In addition, the predicted imputation data can be adjusted for accuracy using GP value. It would get the fewer SNPs in the higher the accuracy. Accuracy and number of variants were negatively correlated. We observed fewer variants under conditions involving higher accuracy. Therefore, decisions about whether to prioritize quantity and accuracy depend on the research [40]. Although we choose GP greater than 0.9, there will be a 10% error rate. Based on an article in Nature Reviews Methods Primers, Uffelmann et al. recommended to remove SNPs less than 0.7(Info Score) [41]. This standard is lower than the 0.9 which we set. In summary, our study compared four imputation algorithms. Our results are freely available for others to use in selecting suitable algorithms for their own research purposes. We also provide a complete SNP database for GWAS and PRS researchers.

## Supplementary Information







## Figures and Tables

**Table 1:** Imputation algorithms. Asterisks indicate the best value in this item.

	IMPUTE2 (WE)	IMPUTE2 (W)	IMPUTE4	IMPUTE5	Beagle5.2
Imputation Time (min)	133	8.5	1.68	1.22	0.68^*^
Storage (Gb)	26	23	14.5	1.5	1^*^
Total Imputed Variants	16,298,564^*^	14,757,187	14,763,606	15,548,597	15,471,490
Intersection with WGS	13,218,326	13,208,509	13,212,007	15,471,490	15,471,490^*^
Extra	3,080,238	1,548,678	1,551,599	77,107	NA^*^
Specificity	0.8110	0.8951	0.8949	0.9950	1.0000^*^
Accuracy	0.9973	0.9971	0.9976^*^	0.9873	0.9875
High Quality Variants	13,182,597	13,169,683	13,180,755	15,275,732	15,277,414^*^
