# Supplementary material for: Comparison of multiple imputation algorithms and verification using whole-genome sequencing in the CMUH genetic biobank
Source: Biomedicine (Taipei). 2021 Dec 1;11(4):57–65. doi: 10.37796/2211-8039.1302 (PMC8823485; doi:10.37796/2211-8039.1302)
Supplement: Supplementary file 1 [file bmed-11-04-057-s001.docx]

|  | IMPUTE2 (WE) | IMPUTE2 (W) | IMPUTE4 | IMPUTE5 | Beagle5.2 |
| --- | --- | --- | --- | --- | --- |
| Imputation Time (min) | 133 | 8.5 | 1.68 | 1.22 | 0.68* |
| Storage (Gb) | 26 | 23 | 14.5 | 1.5 | 1* |
| Total Imputed Variants | 16,298,564* | 14,757,187 | 14,763,606 | 15,548,597 | 15,471,490 |
| Intersection with WGS | 13,218,326 | 13,208,509 | 13,212,007 | 15,471,490 | 15,471,490* |
| Extra | 3,080,238 | 1,548,678 | 1,551,599 | 77,107 | NA* |
| Specificity | 0.8110 | 0.8951 | 0.8949 | 0.9950 | 1.0000* |
| Accuracy | 0.9973 | 0.9971 | 0.9976* | 0.9873 | 0.9875 |
| High Quality Variants | 13,182,597 | 13,169,683 | 13,180,755 | 15,275,732 | 15,277,414* |

Table 1 Imputation algorithms. Asterisks indicate the best value in this item.
