# Supplementary material for: Comparison of multiple imputation algorithms and verification using whole-genome sequencing in the CMUH genetic biobank
Source: Biomedicine (Taipei). 2021 Dec 1;11(4):57–65. doi: 10.37796/2211-8039.1302 (PMC8823485; doi:10.37796/2211-8039.1302)
Supplement: Supplementary file 2 [file bmed-11-04-057-s002.docx]

Comparison of Multiple Imputation Algorithms and Verification Using Whole-Genome Sequencing in the CMUH Genetic Biobank

Ting-Yuan Liu^1^, Chih-Fan Lin^2^, Hsing-Tsung Wu^2^, Ya-Lun Wu^2^, Yu-Chia Chen^1^, Chi-Chou Liao^1^, Yu-Pao Chou^1^, Dysan Chao^1^, Ya-Sian Chang^1,5^, Hsing-Fang Lu^6^, Jan-Gowth Chang^1,5^, Kai-Cheng Hsu^2,3,4*^, Fuu-Jen Tsai^7,8,9,10*^

1. Center for Precision Medicine, China Medical University Hospital, Taichung, 40447, Taiwan.
2. Artificial Intelligence Center for Medical Diagnosis, China Medical University Hospital, Taichung, 40447, Taiwan.
3. Department of Medicine, China Medical University, Taichung, Taiwan
4. Department of Neurology, China Medical University Hospital, Taichung, Taiwan
5. Epigenome Research Center, China Medical University Hospital, Taichung, 40447, Taiwan.
6. Million-person precision medicine initiative, China Medical University Hospital, Taichung, 40447, Taiwan.
7. Department of Medical Research, China Medical University Hospital, Taichung, 40402, Taiwan.
8. School of Chinese Medicine, China Medical University, Taichung, 40402, Taiwan.
9. Division of Pediatric Genetics, Children's Hospital of China Medical University, Taichung, 40447, Taiwan.
10. Department of Biotechnology and Bioinformatics, Asia University, Taichung, 41354, Taiwan.

*corresponding author: Fuu-Jen Tsai

E-mail address: d0704@mail.cmuh.org.tw

Postal address: Department of Medical Research, No. 2, Yude Road, North District, Taichung City, 40447, Taiwan, R.O.C.

*corresponding author: Kai-Cheng Hsu

E-mail address: D35842@mail.cmuh.org.tw

Postal address: Artificial Intelligence Center for Medical Diagnosis, No. 2, Yude Road, North District, Taichung City, 40447, Taiwan, R.O.C.

Key word ：Imputation, SNP Array, Whole Genome Sequencing, CMUH Genetic Biobank
