# Supplementary material for: Comparison of multiple imputation algorithms and verification using whole-genome sequencing in the CMUH genetic biobank
Source: Biomedicine (Taipei). 2021 Dec 1;11(4):57–65. doi: 10.37796/2211-8039.1302 (PMC8823485; doi:10.37796/2211-8039.1302)
Supplement: Supplementary file 3 [file bmed-11-04-057-s003.docx]

Abstract

A genome-wide association study (GWAS) can be conducted to systematically analyze the contributions of genetic factors to a wide variety of complex diseases. Nevertheless, existing GWASs have provided highly ethnic specific data. Accordingly, to provide data specific to Taiwan, we established a large-scale genetic database in a single medical institution at the China Medical University Hospital. With current technological limitations, microarray analysis can detect only a limited number of single-nucleotide polymorphisms (SNPs) with a minor allele frequency of >1%. Nevertheless, imputation represents a useful alternative means of expanding data. In this study, we compared four imputation algorithms in terms of various metrics. We observed that among the compared algorithms, Beagle5.2 achieved the fastest calculation speed, smallest storage space, highest specificity, and highest number of high-quality variants. We obtained 15,277,414 high-quality variants in 175,871 people by using Beagle5.2. In our internal verification process, Beagle5.2 exhibited an accuracy rate of up to 98.75%. We also conducted external verification. Our imputed variants had a 79.91% mapping rate and 90.41% accuracy. These results will be combined with clinical data in future research. We have made the results available for researchers to use in formulating imputation algorithms, in addition to establishing a complete SNP database for GWAS and PRS researchers. We believe that these data can help improve overall medical capabilities, particularly precision medicine, in Taiwan.
